# Davenport’s Diseases of Women

**Published:** 1902-07

**Authors:** 


DAVENPORT'S DISEASES OF WOMEN. A Manual of Gynecology for the use of students and general practitioners. By F. H. Davenport, A. B., M. D., Assistant Professor in Gynecology, Harvard Medical School. New (4th) edition, revised and enlarged in one 12mo volume of 402 pages, with 154 illustrations. Cloth, $1.75, net. Lea Brothers & Co., Publishers. Philadelphia and New York. 1902.

This compact volume describes in terms easily understood the best approved methods for the examination and treatment of all the ordinary diseases of the female pelvic organs. A specialist should of course have at hand more elaborate treatises, but the general practitioner in his every-day work will probably never have need for any more facts bearing upon gynecology than are contained in this work. As a practical guide to both diagnosis and treatment, it is reliable and it possesses the great advantage of being so arranged that it may be easily consulted. R.

